# Resolutions on the Death of Dr. A. L. Talbert

**Published:** 1884-01

**Authors:** T. D. Kelley


### Resolutions on the Death of Dr. A. S. Talbert.

At a meeting of the dentists of the city of Lexington yesterday afternoon at the office of Dr. Driggs, the following resolutions were passed:

WHEREAS, In the midst of life we are called upon to record, with feelings of sorrow, the death of our friend and confrere, Dr. A. S. Talbert; and,

WHEREAS, We humbly submit to that Power which doeth all things well; therefore,

*Resolved*, That we deem it fitting to thus express our condolence over the sad and sudden loss we have sustained.

*Resolved*, That in Dr. Talbert we recognized all the qualities of mind and heart that endeared him to his many friends of this community, and doubly endeared him to the members of his calling, in which latter he labored so long and so faithfully.

*Resolved*, That although he passed away in the fullness of years allotted to men in this life, yet the places of such men are not easily filled, and we will miss with regret that open, honest face and the gentle, yet warm salutation of our departed fellow-laborer.

*Resolved*. That we extend our sympathies and condolence to the family in this their sad bereavement, and that the Secretary be instructed to transmit a copy of these resolutions to the family, and to the various dental journals, and our city papers.

S. DRIGGS,

A. O. RAWLS,

D. L. PROCTOR, } *Committee.*

T. D. KELLEY, *Secretary.*

